# A Modern Syllogistic Method in Intuitionistic Fuzzy Logic with Realistic Tautology

**DOI:** 10.1155/2015/327390

**Published:** 2015-08-24

**Authors:** Ali Muhammad Rushdi, Mohamed Zarouan, Taleb Mansour Alshehri, Muhammad Ali Rushdi

**Affiliations:** ^1^Department of Electrical and Computer Engineering, Faculty of Engineering, King Abdulaziz University, P.O. Box 80204, Jeddah 21589, Saudi Arabia; ^2^Department of Biomedical and Systems Engineering, Cairo University, Giza 12613, Egypt

## Abstract

The Modern Syllogistic Method (MSM) of propositional logic ferrets out from a set of premises *all* that can be concluded from it in the most compact form. The MSM combines the premises into a single function equated to 1 and then produces the complete product of this function. Two fuzzy versions of MSM are developed in Ordinary Fuzzy Logic (OFL) and in Intuitionistic Fuzzy Logic (IFL) with these logics augmented by the concept of Realistic Fuzzy Tautology (RFT) which is a variable whose truth exceeds 0.5. The paper formally proves each of the steps needed in the conversion of the ordinary MSM into a fuzzy one. The proofs rely mainly on the successful replacement of logic 1 (or ordinary tautology) by an RFT. An improved version of Blake-Tison algorithm for generating the complete product of a logical function is also presented and shown to be applicable to both crisp and fuzzy versions of the MSM. The fuzzy MSM methodology is illustrated by three specific examples, which delineate differences with the crisp MSM, address the question of validity values of consequences, tackle the problem of inconsistency when it arises, and demonstrate the utility of the concept of Realistic Fuzzy Tautology.

## 1. Introduction

Fuzzy deductive reasoning has typically relied on a fuzzification of the Resolution Principle of Robinson [1] in first-order predicate calculus. This principle uses a set of premises to prove the validity of a single clause or consequent at a time via the refutation (REDUCTIO AD ABSURDUM) method. Lee [2] proved that a set of clauses is unsatisfiable in fuzzy logic if and only if it is unsatisfiable in two-valued logic. He also proved that if the least truthful clause of a set of clauses has a truth value *a* > 0.5, then all the logical consequents obtained by repeatedly applying the resolution principle have truth values that are never less than *a*. Later, the so-called Mukaidono Fuzzy Resolution Principle, developed by a group of Japanese researchers [3–6], was used to establish a powerful fuzzy Prolog system. The introduction of this principle involved several new concepts, including that of the contradictory degree cd(*X*_*i*_) of a contradiction $\left(X_{i}\wedge {\overset{-}{X}}_{i}\right)$ whose truth value *T*(cd(*X*_*i*_)) equals the truth value $T(X_{i}\wedge {\overset{-}{X}}_{i})$ of the contradiction itself. Recently, a new fuzzy resolution principle was introduced in [7–9], wherein refutation is achieved by the* antonym* not by negation, and reasoning is made more flexible thanks to the existence of a meaningless range, which is a special set that is not true and also not false. Other notable work on various aspects and techniques of fuzzy reasoning and inference is available in [10–23].

The purpose of this paper is to implement fuzzy deductive reasoning via fuzzification of a powerful deductive technique of propositional logic, called the Modern Syllogistic Method (MSM). This method was originally formulated by Blake [24], expounded by Brown [25], and further described or enhanced in [26–33] and has a striking similarity with the resolution-based techniques of predicate logic [1, 34, 35].

The MSM has the distinct advantage that it ferrets out from a set of premises* all* that can be concluded from it, with the resulting conclusions cast in the* simplest* or most compact form. The MSM uses just a single rule of inference, rather than the many rules of inference conventionally employed in propositional-logic deduction (see, e.g., [36, 37]). In fact, the MSM includes all such rules of inference as special cases [30, 31]. The MSM strategy is to convert the set of premises into a single equation of the form *f* = 0 or *g* = 1 and obtain CS(*f*) = the complete sum of *f* (or CP(*g*) = the complete product of *g*). The set of all possible prime consequents of the original premises are obtained from the final equation CS(*f*) = 0 (or CP(*g*) = 1).

We describe herein a fuzzy version of the MSM that utilizes concepts of the Intuitionistic Fuzzy Logic (IFL) [38–48] developed mainly by Atanassov [38, 40, 41, 43–45]. This fuzzy MSM reduces to a restricted version in the Ordinary Fuzzy Logic (OFL) of Zadeh [34, 49–55]. The IFL version of the MSM is more flexible, while the OFL version is simpler and computationally faster. We managed to adapt the MSM to fuzzy reasoning without any dramatic changes of its main steps. In particular, our algorithm for constructing the complete product (or complete sum) of a logic function via consensus generation and absorption remains essentially the same. This algorithm was first developed by Blake [24] and later by Tison [56–59]. It is usually referred to as the Tison method, but we will name it herein as the Blake-Tison method or algorithm.

The organization of the rest of this paper is as follows.  Section 2 briefly reviews the concept of Intuitionistic Fuzzy Logic (IFL) and asserts why it adds necessary flexibility to Ordinary Fuzzy Logic (OFL).  Section 3 combines ideas from Lee [2] and Atanassov [38, 40, 41, 43–45] to produce a novel simple concept of a Realistic Fuzzy Tautology (RFT) and explains why such a new concept is needed.  Section 4 outlines the steps of MSM in two-valued Boolean logic and then adapts it to realistic fuzzy logic, which is an IFL in which the new RFT concept is embedded. Formal proofs of the correctness of this adaptation are provided. Three examples are given in Section 5 to demonstrate the computational steps and to demonstrate how, similar to the result of Lee [2], the validity of the least truthful premise sets an upper limit on the validity of every logical consequent.  Section 6 concludes the paper. The Appendix provides a description of an improved version of the Blake-Tison algorithm for producing the complete product of a logical function.

## 2. Review of Intuitionistic Fuzzy Logic

In Intuitionistic Fuzzy Logic (IFL), a variable *X*_*i*_ is represented by its validity which is the ordered couple

(1) $$\begin{array}{c} V\left(\;X_{i}\;\right)=\langle \;a_{i},b_{i}\;\rangle , \end{array}$$

where *a*_*i*_ and *b*_*i*_ are degrees of truth and falsity of *X*_*i*_, respectively, such that each of the real numbers *a*_*i*_, *b*_*i*_, *a*_*i*_ + *b*_*i*_ ∈ [0,1].

Note that when *a*_*i*_ + *b*_*i*_ = 1, then IFL reduces to Ordinary Fuzzy Logic (OFL), in which *a*_*i*_ alone suffices as a representation for *X*_*i*_, since *b*_*i*_ is automatically determined by *b*_*i*_ = 1 − *a*_*i*_. The necessity of allowing the condition {(*a*_*i*_ + *b*_*i*_) ≤ 1} is established on the grounds that it allows a degree of hesitancy, ignorance, or uncertainty when one can neither designate a variable as true nor label it as false.

Since IFL includes OFL as a special case, operations in IFL should be defined such that they serve as extensions to their OFL counterparts. However, this allows the existence of many definitions for pertinent operations, such as the negation operation [45] or the implication operation [43]. We will stick herein to the most familiar definitions. We have a single unary operation, namely, the negation operation, which produces the complement ${\overset{-}{X}}_{i}$ of a variable *X*_*i*_. We define this operation as one that interchanges the truth and falsity of the variable, that is,

(2) $$\begin{array}{c} V\left(\;{\overset{-}{X}}_{i}\;\right)=\langle \;b_{i},a_{i}\;\rangle . \end{array}$$

The most important binary operations are

(i) the intuitionistic* conjunction* or* meet* operation (*X*_1_∧*X*_2_) defined by
  (3) $$\begin{array}{c} V\left(\;X_{1}\wedge X_{2}\;\right)=\langle \;\min\left(\;a_{1},a_{2}\;\right),\max\left(\;b_{1},b_{2}\;\right)\;\rangle , \end{array}$$
(ii) the intuitionistic* disjunction* or* join* operation (*X*_1_∨*X*_2_) defined by
  (4) $$\begin{array}{c} V\left(\;X_{1}\vee X_{2}\;\right)=\langle \;\max\left(\;a_{1},a_{2}\;\right),\min\left(\;b_{1},b_{2}\;\right)\;\rangle , \end{array}$$
(iii) the intuitionistic* implication* operation $\left(X_{1}\to X_{2})\equiv ({\overset{-}{X}}_{1}\vee X_{2}\right)$ defined herein by
  (5) $$\begin{array}{c} V\left(\;X_{1}⟶X_{2}\;\right)=\langle \;\max\left(\;b_{1},a_{2}\;\right),\min\left(\;a_{1},b_{2}\;\right)\;\rangle . \end{array}$$

With any three intuitionistic fuzzy variables *X*_1_, *X*_2_, and *X*_3_, the following pairs of dual theorems are satisfied:

(1) idempotency:
  (6) X1∨X1=X1,X1∧X1=X1,
(2) commutativity:
  (7) X1∨X2=X2∨X1,X1∧X2=X2∧X1,
(3) associativity:
  (8) X1∨X2∨X3=X1∨X2∨X3,X1∧X2∧X3=X1∧X2∧X3,
(4) absorption:
  (9) X1∨X1∧X2=X1,X1∧X1∨X2=X1,
(5) distributivity:
  (10) X1∨X2∧X3=X1∨X2∧X1∨X3,X1∧X2∨X3=X1∧X2∨X1∧X3,
(6) identities:
  (11) X1∨0=X1,X1∧1=X1.

Atanassov [38, 41] defined the notion of Intuitionistic Fuzzy Tautology (IFT) by the following: *X* is an IFT if and only if *a* ≥ *b*. For comparison, *X* will be a tautology in crisp Boolean algebra if and only if *a* = 1 and *b* = 0.

A variable *X*_1_ is said to be less valid (less truthful) than another variable *X*_2_ (written *V*(*X*_1_) ≤ *V*(*X*_2_)) if and only if *a*_1_ ≤ *a*_2_ and *b*_1_ ≥ *b*_2_. Hence, the complement of an IFT is less valid than this IFT.

## 3. Realistic Fuzzy Tautology

Since our attempts to fuzzify the MSM using the concept of Intuitionistic Fuzzy Tautology (IFT) were not successful, we were obliged to introduce a new concept of tautology that we call Realistic Fuzzy Tautology (RFT). A variable *X*_*i*_ in IFT is an RFT if and only if (*a*_*i*_ > 0.5). Note that an RFT is a more strict particular case of an IFT. If *b*_*i*_ = 1 − *a*_*i*_, then the concept of an RFT reduces to the representation of Fuzzy Tautology given by Lee [2]. A variable *X*_*i*_ in IFT is a non-RFT (denoted by nRFT) if and only if (*a*_*i*_ ≤ 0.5). Hence, two complementary variables *X*_*i*_ and ${\overset{-}{X}}_{i}$ cannot be RFTs at the same time. The conjunction of two complementary variables is nRFT. If the disjunction of a variable with an nRFT is an RFT, then this variable is an RFT. For convenience, we will call the Intuitionistic Fuzzy Logic (IFL) with the concept of RFT embedding in it a Realistic Fuzzy Logic (RFL). The introduction of the RFT concept is utilized herein in fuzzifying the MSM, but it might have other far-reaching consequences in fuzzifying other topics.

## 4. The Modern Syllogistic Method

In this section, we describe the steps of a powerful technique for deductive inference, which is called “the Modern Syllogistic Method” (MSM). The great advantage of the method is that it ferrets out from a given set of premises all that can be concluded from this set, and it casts the resulting conclusions in the simplest or most compact form [24–33].

First, we describe the steps of the MSM in conventional Boolean logic. Then, we adapt these steps to realistic fuzzy logic. Since the MSM has two dual versions, one dealing with propositions equated to zero and the other dealing with propositions equated to one, we are going herein to represent the latter version which corresponds to tautologies.

### 4.1. The MSM in Conventional Boolean Logic

The MSM has the following steps.

**Step 1 .**

Each of the premises is converted into the form of a formula equated to 1 (which we call an equational form), and then the resulting equational forms are combined together into a single equation of the form *g* = 1. If we have *n* logical equivalence relations of the form

(12) $$\begin{array}{c} T_{i}\equiv Q_{i},\;1\leq i\leq n, \end{array}$$

they are set in the equational form

(13) $$\begin{array}{c} P_{i}=\left(\;{\overset{-}{T}}_{i}\vee Q_{i}\;\right)\wedge \left(\;T_{i}\vee {\overset{-}{Q}}_{i}\;\right),\;1\leq i\leq n. \end{array}$$

We may also have (*m* − *n*) logical implication (logical inclusion) relations of the form

(14) $$\begin{array}{c} T_{i}⟶Q_{i},\;\left(n+1\right)\leq i\leq m. \end{array}$$

These relations symbolize the statements “If *T*_*i*_ then *Q*_*i*_” or equivalently “*T*_*i*_ if only *Q*_*i*_”. Conditions (14) can be set into the equational form

(15) $$\begin{array}{c} P_{i}={\overset{-}{T}}_{i}\vee Q_{i}=1,\;\left(n+1\right)\leq i\leq m. \end{array}$$

**Step 2 .**

The totality of premises in (13) and (15) finally reduces to the single equation *g* = 1, where *g* is given by

(16) g⋀i=1mPi=⋀i=1nT−i∨Qi∧Ti∨Q−i∧⋀i=n+1mT−i∨Qi.

Equations (13) and (15) represent the dominant forms that premises can take. Other less important forms are discussed by Klir and Marin [60] and can be added to (16) when necessary.

**Step 3 .**

The function *g* in (16) is rewritten as a complete product (a dual Blake canonical form), that is, as a conjunction of all the prime implicates of *g*. There are many manual and computer algorithms for developing the complete product of a switching function [25]. Most of these algorithms depend on two logical operations: (a) consensus generation and (b) absorption.

**Step 4 .**

Suppose the complete product of *g* takes the form

(17) $$\begin{array}{c} CP\left(\;g\;\right)=\overset{l}{\underset{i=1}{⋀}}C_{i}=1, \end{array}$$

where *C*_*i*_ is the *i*th prime implicate of *g*. Equation (17) is equivalent to the set of equations

(18) $$\begin{array}{c} C_{i}=1,\;1\leq i\leq l. \end{array}$$

Equations (18) are called prime consequents of *g* = 1 and state in the simplest equational form all that can be concluded from the original premises. The conclusions in (18) can also be cast into implication form. Suppose *C*_*i*_ is given by a disjunction of complemented literals ${\overset{-}{X}}_{ij}$ and uncomplemented literals *Y*_*ij*_, that is,

(19) $$\begin{array}{c} C_{i}=\overset{r}{\underset{j=1}{⋁}}\;{\overset{-}{X}}_{ij}\vee \overset{s}{\underset{j=1}{⋁}}Y_{ij},\;1\leq i\leq l, \end{array}$$

then (18) can be rewritten as

(20) $$\begin{array}{c} \overset{r}{\underset{j=1}{⋀}}X_{ij}⟶\overset{s}{\underset{j=1}{⋁}}Y_{ij},\;1\leq i\leq l, \end{array}$$

### 4.2. The MSM in Realistic Fuzzy Logic

A crucial prominent feature of realistic fuzzy logic is that it can be used to implement the MSM without spoiling any of its essential features. We just need to replace the concept of a crisp logical “1” by that of the realistic fuzzy tautology (RFT) introduced in Section 3. Now, a realistic fuzzy version of the MSM has the following steps.

**Step 1 .**

Assume the problem at hand is governed by a set of RFTs *P*_*i*_, 1 ≤ *i* ≤ *n*. Each of these RFTs might be assumed from the outset or be constructed from equivalence or implication relations. Let *P*_*i*_ be described by

(21) $$\begin{array}{c} V\left(\;P_{i}\;\right)=\langle \;\mu_{i},\gamma_{i}\;\rangle . \end{array}$$

**Step 2 .**

The given set of RFT premises are equivalent to the single function

(22) g=⋀imPi,Vg=min i⁡μi,max i⁡γi.

The function *g* is also an RFT. This equivalence is proved in Theorem 1.

**Step 3 .**

Replace the function *g* by its complete product CP(*g*). The resulting CP(*g*) is also an RFT since the operations used in going from *g* to CP(*g*) preserve the RFT nature. These operations are as follows:

- absorption, which is known to be tautology-preserving in general fuzzy logic and intuitionistic fuzzy logic and hence in the current realistic fuzzy logic,
- consensus generation, which preserves RFTs in the sense that when the conjunction of two clauses is an RFT, then it remains so when conjuncted with the consensus of these two clauses. This is proved in the form of Theorem 2.

**Step 4 .**

Since CP(*g*) is an RFT, then when it is given by the conjunction in (17), each clause *C*_*i*_, 1 ≤ *i* ≤ *l*, in (17) will be an RFT (again thanks to Theorem 1). The fact that each of the clauses *C*_*i*_ is an RFT is all that can be concluded from the original premises. The procedure does not necessarily provide specific information about the validity of each consequent *C*_*i*_. However, as we show in the examples below, it is possible to obtain such information in specific cases.

**Theorem 1 .**

Each of the realistic fuzzy variables *P*_*i*_, 1 ≤ *i* ≤ *m* is an RFT if and only if their conjunction ⋀_*i*=1_^*m*^ *P*_*i*_ is an RFT.

**Proof**

Consider the following:

(23) Pi  is  an  RFT,  1≤i≤m⟺μi>0.5,  1≤i≤m⟺min i⁡μi>0.5⟺⋀i=1mPi  is  an  RFT.

**Theorem 2 .**

The conjunction of two clauses with a single opposition retains the RFT property when augmented by a third clause representing the consensus of the two original clauses. Specifically, if $\left(X_{1}\vee X_{2})\wedge ({\overset{-}{X}}_{1}\vee X_{3}\right)$ is an RFT, then $\left(X_{1}\vee X_{2})\wedge ({\overset{-}{X}}_{1}\vee X_{3})\wedge (X_{2}\vee X_{3}\right)$ is also an RFT.

**Proof**

Let *V*(*X*_*i*_) = 〈*a*_*i*_, *b*_*i*_〉, *i* = 1,2, 3. By virtue of Theorem 1, the fact that $\left(X_{1}\vee X_{2})\wedge ({\overset{-}{X}}_{1}\vee X_{3}\right)$ is an RFT implies that (*X*_1_∨*X*_2_) is an RFT (i.e., max⁡(*a*_1_, *a*_2_) > 0.5) and that $\left({\overset{-}{X}}_{1}\vee X_{3}\right)$ is an RFT (i.e., max⁡(*b*_1_, *a*_3_) > 0.5).

Now consider two cases.

*Case 1*. One has {*a*_1_ ≥ 0.5}⇒{*b*_1_ ≤ 0.5}, and hence

(24) max⁡b1,a3>0.5a3>0.5maxa2,a3>0.5⟹X2∨X3  is  an  RFT.

*Case 2*. One has *a*_1_ ≤ 0.5

(25) a1≤0.5∧max⁡a1,a2>0.5⟹a2>0.5⟹max⁡a2,a3>0.5⟹X2∨X3  is  an  RFT.

Now each of (*X*_1_∨*X*_2_), $\left({\overset{-}{X}}_{1}\vee X_{3}\right)$, and (*X*_2_∨*X*_3_) is an RFT. Hence, thanks to Theorem 1, their conjunction $\left(X_{1}\vee X_{2})\wedge ({\overset{-}{X}}_{1}\vee X_{3})\wedge (X_{2}\vee X_{3}\right)$ is an RFT.

One prominent difference between fuzzy MSM and ordinary MSM is that the complementary laws

(26) Xi∨X−i=1Xi∧X−i=0

in ordinary logic do not hold in any fuzzy logic including OFL, IFL, or RFL. This means that in implementing our algorithm for generating the complete product of a switching function, a conjunction of the form $\left(X_{i}\wedge {\overset{-}{X}}_{i}\right)$ might appear, and then it is left as it is, and not replaced by 0. This point will be clarified further in Example 2 of Section 5.

Table 1 employs the MSM to derive fuzzy versions of many famous rules of inference, including, in particular, the celebrated rules of MODUS PONENS and MODUS TOLLENS. The derivation shows that some of the rules have some intermediate consequences as well as a final particular consequence.

## 5. Examples

**Example 1 .**

A typical example of MSM presented by Brown [25], pp. 124–127, and taken from Kalish and Montague [61], has the following statements:

(1) if Alfred studies, then he receives good grades (*S* → *G*);
(2) if Alfred does not study, then he enjoys college ($\overset{-}{S}\to E$);
(3) if Alfred does not receive good grades, then he does not enjoy college ($\overset{-}{G}\to \overset{-}{E}$).

The MSM solution combines the above premises into a single equation

(27) $$\begin{array}{c} g_{1}=\left(\;\overset{-}{S}\vee G\;\right)\wedge \left(\;S\vee E\;\right)\wedge \left(\;G\vee \overset{-}{E}\;\right)=1 \end{array}$$

and obtains the complete product of *g*_1_ by adding consensus alterms or clauses [56] with respect to the biform variables *S* and *E* and absorbing subsuming alterms (see Appendix). Gradually, the formula for *g*_1_ changes to end up as the complete product form:

(28) g1S−∨G∧S∨E∧G∨E−∧G∨E=S−∨G∧S∨E∧G∨E−∧G∨E∧S∨G∧GS∨E∧G.

The last expression for *g*_1_ is CP(*g*_1_) and is still equated to 1. Hence, it asserts the not so-obvious conclusion of (*G* = 1) {Alfred  receives  good  grades} beside the conclusion {(*S*∨*E*) = 1}, which is just a reecho of one of the premises. These two conclusions are* all* that can be concluded from the premises in the simplest form. Any other valid conclusion must subsume one of these two conclusions. Now, suppose that our knowledge about the premises is fuzzy or uncertain, so that each of the premises is no longer a crisp tautology, but is weakened to the status of a realistic fuzzy tautology (RFT). To be specific, let us assign the following values for the validity of each premise:

(29) VS⟶GVS−∨G=0.6,0.3,VS−⟶EVS∨E=0.9,0.1,VG−⟶E−VG∨E−=0.8,0.1.

The function *g*_1_ in (27) is no longer a crisp tautology (=1), but rather an RFT with validity

(30) Vg1VS−∨G∧S∨E∧G∨E−=min⁡0.6,0.9,0.8,max⁡0.3,0.1,0.1=0.6,0.3,

so *g*_1_ inherits the validity of the first premise, which is the least-truthful premise. This validity is also inherited by CP(*g*_1_) in the last line of  (28) and also by the novel consequent (*G* = 1), that is,

(31) $$\begin{array}{c} V\left(\;G\;\right)=\langle \;0.6,0.3\;\rangle . \end{array}$$

This means that the consequent {Alfred  gets  good  grades} has a truth value of 0.6 and a falsity value of 0.3. The fact that (0.6 + 0.3) = 0.9 < 1 leaves room for our uncertainty or ignorance about this fuzzy proposition.

**Example 2 .**

The MSM has a built-in capability of detecting inconsistency in a set of premises, since this produces CP(*g*) as 0, and leads to {0 = 1} which is unacceptable in two-valued logic [30, 31]. This feature is still enjoyed by the fuzzy MSM since an inconsistency will be revealed in the form of a variable and its complement being both RFT, which is a contradiction. For a specific example, consider the set of premises $\left(A↔\overset{-}{B}\right)$, $\left(B↔\overset{-}{C}\right)$, and $\left(C↔\overset{-}{A}\right)$. In equational form, these reduce to

(32) A−∨B−∧A∨B=1,B−∨C−∧B∨C=1,C−∨A−∧C∨A=1,

or equivalently to the single equation

(33) g2A−∨B−∧A∨B∧B−∨C−∧B∨C∧C−∨A−∧C∧A=1.

In two-valued logic, the complete product of *g*_2_ is obtained via the Improved Blake-Tison Method (see Appendix) as

(34) $$\begin{array}{c} CP\left(\;g_{2}\;\right)=A\wedge \overset{-}{A}\wedge B\wedge \overset{-}{B}\wedge C\wedge \overset{-}{C}=0, \end{array}$$

which leads to the contradiction (0 = 1). However, in realistic fuzzy logic, we have

(35) $$\begin{array}{c} CP\left(\;g_{2}\;\right)=A\wedge \overset{-}{A}\wedge B\wedge \overset{-}{B}\wedge C\wedge \overset{-}{C} \end{array}$$

being an RFT. This means that both *A* and $\overset{-}{A}$ (and also both *B* and $\overset{-}{B}$ and both *C* and $\overset{-}{C}$) are RFTs, which is a contradiction. Hence, the original set of premises are inconsistent.

**Example 3 .**

Consider the set of premises [30, 37]:

(1) Pollution will increase if government restrictions are relaxed (*R* → *P*).
(2) If pollution increases, there will be a decline in the general health of the population (*P* → *D*).
(3) If there is a decline in health in the population, productivity will fall (*D* → *F*).
(4) The economy will remain healthy only if productivity does not fall $\left(E\to \overset{-}{F}\right)$.

These premises are equivalent to the propositional equation (*g*_3_ = 1), where

(36) $$\begin{array}{c} g_{3}=\left(\;\overset{-}{R}\vee P\;\right)\wedge \left(\;\overset{-}{P}\vee D\;\right)\wedge \left(\;\overset{-}{D}\vee F\;\right)\wedge \left(\;\overset{-}{E}\vee \overset{-}{F}\;\right). \end{array}$$

The complete product of *g*_3_ is obtained via the Improved Blake-Tison Method (see Appendix) as

(37) CPg3=R−∨P∧P−∨D∧D−∨F∧E−∨F−∧R−∨D∧D−∨E−∧P−∨F∧P−∨E−∧R−∨F∧R−∨E−.

The fact that CP(*g*_3_) = 1 means that there are six new consequents (that are not just a reecho of premises). The last of these consequents is

(38) $$\begin{array}{c} \overset{-}{R}\vee \overset{-}{E}=1, \end{array}$$

or equivalently

(39) $$\begin{array}{c} R⟶\overset{-}{E}, \end{array}$$

which means that if government restrictions are relaxed, then the economy will not remain healthy, an argument in favor of a stronger governmental regulatory role.

Now, suppose that the given premises are not crisp tautologies, but are just RFTs with respective validities

(40) VR⟶PVR−∨P=0.6,0.3,VP⟶DVP−∨D=0.7,0.2,VD⟶FVD−∨F=0.8,0.1,VE⟶F−VE−∨F−=0.9,0.1.

Hence, each of the new clauses in (37) is an RFT of a validity dependent on the validities of the clauses generating it.  Table 2 lists these new clauses, identifies their generators, and hence assigns a validity to each of them. The issue of a stronger regulatory role for the government now has a validity of 〈0.6,0.3〉 rather than 〈1.0,0.0〉. This validity is realistic in the sense that this issue can be viewed as supported by 60% of the voters and opposed by 30% of them, with 10% of them abstaining or undecided.

## 6. Conclusion

The Modern Syllogistic Method (MSM) is a sound and complete single rule of inference that encompasses all rules of inference. It extracts from a given set of premises all that can be concluded from it in the simplest possible form. It has a striking similarity with resolution-based techniques in predicate logic, but while these techniques chain* backwardly* from a given assertion seeking to* refute* it, the MSM chains* forwardly* from the set of premises seeking to* prove all* possible consequences [25].

This paper contributes a fuzzy version of MSM using a variant of Intuitionistic Fuzzy Logic (IFL) called Realistic Fuzzy Logic (RFL). Here, a propositional variable is characterized by 2-tuple validity expressing its truth and falsity. Automatically, a third dependent attribute for the variable emerges, namely, hesitancy or ignorance about the variable, which complements the sum of truth and falsity to 1. If Ignorance is 0, then IFL reduces to Ordinary Fuzzy Logic (OFL) and the RFL version of MSM reduces to a simpler but weaker OFL version. The slight restriction of IFL to RFL involves the replacement of the concept of an Intuitionistic Fuzzy Tautology (IFT), in which truth is greater than or equal to falsity, by a restricted concept of Realistic Fuzzy Tautology (RFT) in which truth is* strictly* greater than 0.5. The introduction of the RFT enabled us to fuzzify the MSM without making any significant changes in it and to formally prove the correctness of all the steps of the emergent fuzzy MSM. As an offshoot, the paper contributes an improvement of the main algorithm that constitutes the heart of the MSM, whether it is crisp, ordinary fuzzy, or realistic fuzzy. The improvement involves a matrix formulation of the typical step of consensus generation that minimizes the comparisons among pairs of alterms that might have consensus alterms. The following task of absorbing subsuming alterms is also reduced considerably via a set of novel observations that were formally proved. The concept of consensus used herein is exactly the one used in crisp two-valued propositional logic. There was no need herein to introduce a specific concept of fuzzy consensus. The only significant change is that relations (26) no longer hold.

The fuzzy MSM methodology is illustrated by three specific examples, which delineate differences with the crisp MSM, address the question of validity values of consequences, tackle the problem of inconsistency when it arises, and demonstrate the utility of RFL compared to ordinary fuzzy logic.

The current paper is one of several new papers by the authors which are intended to extend the utility and sharpen the mathematics of the MSM. One of these papers [62] presents an incremental version of the MSM, in which the core work of the MSM is not completely repeated but is slightly incremented when additional premises are added. Another paper [63] utilizes the MSM in the exploration of hidden aspects in engineering ethical dilemmas by investigating different scenarios describing the situation from various perspectives.

In future work, we hope to combine the contributions of the current paper with those of [62, 63]. We also hope to utilize the new RFT concept introduced herein in novel applications.

## Figures and Tables

**Figure 1:**
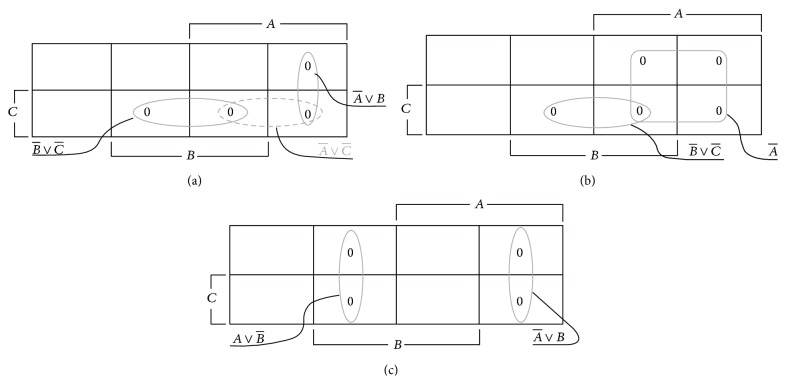
(a) The alterms $\left(\overset{-}{A}\vee B\right)$ and $\left(\overset{-}{B}\vee \overset{-}{C}\right)$ have a single opposition (disjoint loops sharing a border), and hence their conjunction can be augmented by their consensus $\left(\overset{-}{A}\vee \overset{-}{C}\right)$. (b) The alterms $\overset{-}{A}$ and $\left(\overset{-}{B}\vee \overset{-}{C}\right)$ have zero opposition (nondisjoint or overlapping) loops, and hence no consensus (or a consensus of 1). (c) The alterms $\left(\overset{-}{A}\vee B\right)$ and $\left(A\vee \overset{-}{B}\right)$ have more than one opposition (disjoint faraway loops) and have no consensus (or a consensus of 1).

**Table 1:** MSM derivation of fuzzy versions of famous rules of inference {the particular conclusion of a rule is highlighted in bold}.

Rule name	Fuzzy RFT antecedents (premises)	Premises as separate fuzzy equations *P*_*i*_ = RFT, *i* = 1,…, *m*	Premises as a single fuzzy equation *g* = RFT	Conclusions as a single fuzzy equation CP(*g*) = RFT	Conclusions as separate fuzzy equations *C*_*i*_ = RFT, *i* = 1,…, *l*	Fuzzy RFT consequence (conclusion)
MODUS PONENS	*A* → *B* *A*	$\overset{-}{A}\vee B=\text{RFT}$ *A* = RFT	$\left(\overset{-}{A}\vee B\right)\wedge A=\text{RFT}$	*B*∧*A* = RFT	**B** = **R** **F** **T** *A* = RFT	*B*

MODUS TOLLENS	*A* → *B* $\overset{-}{B}$	$\overset{-}{A}\vee B=\text{RFT}$ $\overset{-}{B}=\text{RFT}$	$\left(\overset{-}{A}\vee B\right)\wedge \overset{-}{B}=\text{RFT}$	$\overset{-}{A}\wedge \overset{-}{B}=\text{RFT}$	$\overset{-}{A}=RFT$ $\overset{-}{B}=\text{RFT}$	$\overset{-}{A}$

HYPOTHETICAL SYLLOGISM	*A* → *B* *B* → *C*	$\overset{-}{A}\vee B=\text{RFT}$ $\overset{-}{B}\vee C=\text{RFT}$	$\left(\overset{-}{A}\vee B\right)\wedge \left(\overset{-}{B}\vee C\right)=\text{RFT}$	$\left(\overset{-}{A}\vee B\right)\wedge \left(\overset{-}{B}\vee C\right)\wedge \left(\overset{-}{A}\vee C\right)=\text{RFT}$	$\overset{-}{A}\vee B=\text{RFT}$ $\overset{-}{B}\vee C=\text{RFT}$ $\overset{-}{A}\vee C=RFT$	*A* → *C*

SIMPLIFICATION	*A*∧*B*	*A*∧*B* = RFT	*A*∧*B* = RFT	*A*∧*B* = RFT	**A** = **R** **F** **T** *B* = RFT	*A*

CONJUNCTION	*A* *B*	*A* = RFT *B* = RFT	*A*∧*B* = RFT	*A*∧*B* = RFT	**A**∧**B** = **R** **F** **T**	*A*∧*B*

CONSTRUCTIVE DILEMMA	*A* → *B* *C* → *D* *A*∨*C*	$\overset{-}{A}\vee B=\text{RFT}$ $\overset{-}{C}\vee D=\text{RFT}$ *A*∨*C* = RFT	$\left(\overset{-}{A}\vee B\right)\wedge \left(\overset{-}{C}\vee D\right)$ ∧(*A*∨*C*) = RFT	$\left(\overset{-}{A}\vee B\right)\wedge \left(\overset{-}{C}\vee D\right)\wedge \left(A\vee C\right)\wedge \left(B\vee C\right)$ ∧(*A*∨*D*)∧(*B*∨*D*) = RFT	$\left(\overset{-}{A}\vee B\right)=\text{RFT}$ $\left(\overset{-}{C}\vee D\right)=\text{RFT}$ (*A*∨*C*) = RFT (*B*∨*C*) = RFT (*A*∨*D*) = RFT (**B**∨**D**) = RFT	*B*∨*D*

DISJUNCTIVE SYLLOGISM	*A*∨*B* $\overset{-}{A}$	*A*∨*B* = RFT $\overset{-}{A}=\text{RFT}$	$\left(A\vee B\right)\wedge \overset{-}{A}=\text{RFT}$	$B\wedge \overset{-}{A}=\text{RFT}$	**B** = **R** **F** **T** $\overset{-}{A}=\text{RFT}$	*B*

ADDITION	*A*	*A* = RFT	*A* = RFT	*A* = RFT	**A**∨**B** = **R** **F** **T** {An alterm subsuming an RFT alterm is also RFT}	*A*∨*B*

ABSORPTION	*A* → *B*	$\overset{-}{A}\vee B=\text{RFT}$	$\overset{-}{A}\vee B=\text{RFT}$	$\overset{-}{A}\vee B=\text{RFT}$	$\overset{-}{A}\vee AB=RFT$ $\left\{\right.\overset{-}{A}\vee AB=\overset{-}{A}\vee B$ by reflection law}	*A* → *AB*

CASES	*A* *A* → (*C*∨*D*) *C* → *B* *D* → *B*	*A* = RFT $\overset{-}{A}\vee C\vee D=\text{RFT}$ $\overset{-}{C}\vee B=\text{RFT}$ $\overset{-}{D}\vee B=\text{RFT}$	$A\wedge \left(\overset{-}{A}\vee C\vee D\right)\wedge \left(\overset{-}{C}\vee B\right)$ $\wedge \left(\overset{-}{D}\vee B\right)=\text{RFT}$	*A*∧*B*∧(*C*∨*D*) = RFT	*A* = RFT **B** = **R** **F** **T** *C*∨*D* = RFT	*B*

CASE ELIMINATION	*A*∨*B* $A\to \left(C\wedge \overset{-}{C}\right)$	*A*∨*B* = RFT $\overset{-}{A}\vee \left(C\wedge \overset{-}{C}\right)=\text{RFT}$	$\left(A\vee B\right)\wedge \left(\overset{-}{A}\vee \left(C\wedge \overset{-}{C}\right)\right)=\text{RFT}$	$\left(A\vee B\right)\wedge \left(\overset{-}{A}\vee \left(C\wedge \overset{-}{C}\right)\right)$ $\wedge \left(B\vee \left(C\vee \overset{-}{C}\right)\right)=\text{RFT}$	*A*∨*B* = RFT $\overset{-}{A}\vee \left(C\wedge \overset{-}{C}\right)=\text{RFT}$ $B\vee \left(C\wedge \overset{-}{C}\right)=\text{RFT}$ {**B** = **R** **F** **T** and $C\wedge \overset{-}{C}=\text{nRFT}\left.\right\}$	*B*

REDUCTIO AD ABSURDUM (CONTRADICTION)	$\overset{-}{A}\to \left(\overset{-}{B}\wedge B\right)$	$A\vee \left(\overset{-}{B}\wedge B\right)=\text{RFT}$	$A\vee \left(\overset{-}{B}\wedge B\right)=\text{RFT}$	$A\vee \left(\overset{-}{B}\wedge B\right)=\text{RFT}$	$A\vee \left(\overset{-}{B}\wedge B\right)=\text{RFT}$ $\left\{A=RFT,\overset{-}{B}\wedge B=\text{nRFT}\right\}$	*A*

**Table 2:** Validities of consequences obtained in Example 3.

New clause	Nature	Validity
$\left(R\to D\right)\equiv \left(\overset{-}{R}\vee D\right)$	Consensus of $\left(\overset{-}{R}\vee P\right)$ and $\left(\overset{-}{P}\vee D\right)$	〈0.6,0.3〉
$\left(D\to \overset{-}{E}\right)\equiv \left(\overset{-}{D}\vee \overset{-}{E}\right)$	Consensus of $\left(\overset{-}{D}\vee F\right)$ and $\left(\overset{-}{E}\vee \overset{-}{F}\right)$	〈0.8,0.1〉
$\left(P\to F\right)\equiv \left(\overset{-}{P}\vee F\right)$	Consensus of $\left(\overset{-}{P}\vee D\right)$ and $\left(\overset{-}{D}\vee F\right)$	〈0.7,0.2〉
$\left(P\to \overset{-}{E}\right)\equiv \left(\overset{-}{P}\vee \overset{-}{E}\right)$	Consensus of $\left(\overset{-}{P}\vee D\right)$ and $\left(\overset{-}{D}\vee \overset{-}{E}\right)$	〈0.7,0.2〉
$\left(R\to F\right)\equiv \left(\overset{-}{R}\vee F\right)$	Consensus of $\left(\overset{-}{R}\vee D\right)$ and $\left(\overset{-}{D}\vee F\right)$	〈0.6,0.3〉
$\left(R\to \overset{-}{E}\right)\equiv \left(\overset{-}{R}\vee \overset{-}{E}\right)$	Consensus of $\left(\overset{-}{R}\vee F\right)$ and $\left(\overset{-}{E}\vee \overset{-}{F}\right)$	〈0.6,0.3〉

**Table 3:** The general layout of the consensus generation table of the Improved Blake-Tison Method when producing consensus alterms with respect to *X*_*m*_. The vertical keys of this table are the alterms containing *X*_*m*_ and its horizontal keys are the alterms containing ${\overset{-}{X}}_{m}$ while alterms containing neither *X*_*m*_ nor ${\overset{-}{X}}_{m}$ are set aside.

		⋯	(*A*_*j*_∨*X*_*m*_)	⋯	(*A*_*k*_∨*X*_*m*_)	⋯

⋮			⋮		⋮	
$\left(S_{i}\vee {\overset{-}{X}}_{m}\right)$		⋯	{*S*_*i*_∨*A*_*j*_}	⋯	{*S*_*i*_∨*A*_*k*_}	⋯
⋮			⋮		⋮	
$\left(S_{r}\vee {\overset{-}{X}}_{m}\right)$		⋯	{*S*_*r*_∨*A*_*j*_}	⋯	{*S*_*r*_∨*A*_*k*_}	⋯
⋮			⋮		⋮	

Set-aside alterms						
(alterms containing neither *X*_*m*_ nor ${\overset{-}{X}}_{m}$)						

## Appendix

### The Improved Blake-Tison Method (ITM)

The complete sum of a switching function *f*, to be denoted by CS(*f*), is the all-prime-implicant disjunction that expresses *f*, that is, it is a sum-of-products (SOP) formula whose products are all the prime implicants of *f*. The complete sum is called the “Blake canonical form” by Brown [25] in honor of *A*. Blake who was the first person to study this form in his thesis [24]. Since CS(*f*) is a disjunction of all the prime implicants of *f*, and nothing else, it is obviously unique and hence stands for a canonical representation of the switching function [25]. The dual quantity of the complete sum is the complete product of a switching function *g*, denoted CP(*g*), which is the all-prime-implicate conjunction that expresses *g*, that is, it is a product-of-sum (POS) formula whose alterms or sums are all the prime implicates of *f* [56].

The concept of the complete product of a switching function *g* is closely related to that of a dual syllogistic formula for *g*. However, while CP(*g*) is unique and canonical, there are infinitely many dual syllogistic formulas for *g*. A dual syllogistic formula of *g* can be defined as a POS formula whose alterms include, but are not necessarily confined to, all the prime implicates of *g*, that is, it is the complete product of *g* conjuncted (possibly) with alterms each of which subsumes some prime implicates of *g*. The complete-product formula CP(*g*) is minimal within the class of dual syllogistic formulas for *g*, that is, the set of alterms in any dual syllogistic formula for *g* is a superset of the set of alterms in CP(*g*). Hence, CP(*g*) can be denoted by ABS(*G*), where *G* is any dual syllogistic formula for *g* and ABS(*G*) denotes an equivalent absorptive formula of *G*, that is, a formula obtained from *G* by successive deletion of alterms absorbed in other alterms of *G*. The complete-product formula CP(*g*) may be generated by the following two-step iterative-consensus procedure: (a) Find a dual syllogistic formula *G* for *g* by continually comparing alterms and adding their consensus alterms to the current formula of *g* and (b) delete absorbed alterms to obtain ABS(*G*). Note that two alterms have a consensus if and only if they have exactly one opposition, that is, exactly one variable that appears complemented in one alterm and appears uncomplemented in the other. In such a case, the consensus is the ORing of the remaining literals of the two alterms, with idempotency of the OR operation being taken into consideration. The concept of a consensus of two alterms is illustrated in Figure 1.

Tison method (see, e.g., [56–59, 64–67]) is a systematic streamlined version of the iterative-consensus technique for obtaining the CS of a switching function *f*, or dually the CP of a switching function *g*. The original study of Tison appeared in [57], but a more readable exposition can be found in [58], and further proofs are available in [58, 59]. Related work and techniques are also available in [68–77]. Since Tison method is actually due to Blake [24], we will present it here under the name Blake-Tison Method. Its essence when used for obtaining the complete product is summarized as follows.

*Blake-Tison Algorithm.* Start with a set of *n*_0_ alterms or sums of literals *s*_0_ = {*A*_1_^(0)^, *A*_2_^(0)^,…, *A*_*n*_0__^(0)^} with biform variables *X*_1_, *X*_2_,…, *X*_*M*_ and a Boolean function *g* that is expressed by conjunction of the alterms in *s*_0_. Assume that any absorbable alterms in *s*_0_ have been deleted, so that the conjunction of alterms in *s*_0_ is an absorptive formula. For 1 ≤ *m* ≤ *M*, repeat the following 2-part step that replaces an absorptive set of alterms *s*_*m*−1_ by another *s*_*m*_:

(1) For 1 ≤ *j* ≤ *k* ≤ *n*_(*m*−1)_, if *X*_*m*_ appears complemented in one of the two alterms *A*_*j*_^(*m*−1)^ and *A*_*k*_^(*m*−1)^ and appears uncomplemented in the other such that the two alterms have no other opposition, then they have a consensus with respect to *X*_*m*_. Form that consensus and add it to *s*_*m*−1_. Finally, *s*_*m*−1_ is replaced by a superset ${\overset{-}{s}}_{m-1}$ of *J*_(*m*−1)_ elements, where *J*_(*m*−1)_ ≥ *n*_(*m*−1)_.
(2) Consider every pair *A*_*j*_^(*m*−1)^, *A*_*k*_^(*m*−1)^, *j* ≠ *k* of (so far remaining) products in ${\overset{-}{s}}_{m-1}$. If *A*_*j*_^(*m*−1)^ subsumes *A*_*k*_^(*m*−1)^, then delete *A*_*j*_^(*m*−1)^. Otherwise, if *A*_*j*_^(*m*−1)^ is subsumed by *A*_*k*_^(*m*−1)^, then delete *A*_*k*_^(*m*−1)^. Whenever all subsumptions (and subsequent deletions) are exhausted, let the remaining absorptive set be *s*_*m*_ = {*A*_1_^(*m*)^, *A*_2_^(*m*)^, *A*_*n*_*m*__^(*m*)^}.

Blake [24] and later Cutler et al. [58] formally proved Theorem 3, asserting the success of the Blake-Tison algorithm in obtaining CP(*g*) by merely applying the iterative-consensus procedure to each biform variable one by one.

Theorem 3 . In the Blake-Tison algorithm above,

(a) the conjunction of alterms in any of the sets *s*_*m*_, where 1 ≤ *m* ≤ *M* is an expression of *g*,
(b) the final set *s*_*M*_ consists of all prime implicates of *g*.

Rushdi and Al-Yahya [64] proposed an improvement of Blake-Tison's Method in which the typical step starts by arranging a given expression for *g* with respect to a biform variable *X*_*m*_, 1 ≤ *m* ≤ *M*, in the form

(A.1) $$\begin{array}{c} g=\left(\;r\vee {\overset{-}{X}}_{m}\;\right)\wedge \left(\;s\vee X_{m}\;\right)\wedge t, \end{array}$$

where *r* = ⋀_*i*=1_^*n*_*r*_^ *r*_*i*_, *s* = ⋀_*j*=1_^*n*_*s*_^ *s*_*j*_, and *t* = ⋀_*k*=1_^*n*_*t*_^ *t*_*k*_ are POS formulas that are independent of *X*_*m*_, and the symbols *r*_*i*_, *s*_*j*_, and *t*_*k*_ denote alterms or sums of single literals. Thanks to intelligent multiplication [25, 64], the function *g* takes the POS form

(A.2) $$\begin{array}{c} g=\overset{n_{r}}{\underset{i=1}{⋀}}\left(\;r_{i}\vee {\overset{-}{X}}_{m}\;\right)\wedge \overset{n_{s}}{\underset{j=1}{⋀}}\left(\;s_{j}\wedge X_{m}\;\right)\wedge \overset{n_{t}}{\underset{k=1}{⋀}}t_{k}. \end{array}$$

Next *g* is augmented by all consensus alterms with respect to *X*_*m*_, which turn out to be the alterms (*r*_*i*_∨*s*_*j*_) which do not add to 1 in the expression

(A.3) $$\begin{array}{c} \overset{n_{r}}{\underset{i=1}{⋀}}\;\overset{n_{s}}{\underset{j=1}{⋀}}\left(\;r_{i}\vee s_{j}\;\right). \end{array}$$

This is followed by absorbing or deleting alterms that subsume others. The method repeats this typical step for all biform variables ending with CP(*g*) after the last step.

Table 3 suggests an economic layout [64] for implementing the typical step in the Improved Blake-Tison Method (IBTM) with a restricted number for the comparisons needed for implementing absorptions. This typical step, which performs consensus generation with respect to a specific biform variable *X*_*m*_, involves a rearrangement of the alterms whose conjunction constitutes the current formula of *g* at this step. We construct a consensus-generation table with respect to *X*_*m*_ that resembles a multiplication table or matrix. The vertical keys of this table are the alterms containing the uncomplemented literal *X*_*m*_ and its horizontal keys are the alterms containing the complemented literal ${\overset{-}{X}}_{m}$, while its entries are the consensus alterms generated by these keys with respect to *X*_*m*_. Alterms containing neither the uncomplemented literal *X*_*m*_ nor the complemented literal ${\overset{-}{X}}_{m}$ are set aside and naturally not included in the consensus generation of the table but might absorb or be absorbed by the consensus alterms produced by the table. Table 3 shows typical keys and entries of the consensus-generation table, where we use the symbol {*S*_*i*_∨*A*_*j*_} to denote the consensus of the vertical key (*A*_*j*_∨*X*_*m*_) with the horizontal key $\left(S_{i}\vee {\overset{-}{X}}_{m}\right)$, which is the ORing of the two alterms *S*_*i*_ and *A*_*j*_ after deleting any repeated literals (thanks to the idempotency of the logical operation “OR”). Of course, if the alterms *S*_*i*_ and *A*_*j*_ have at least one opposition, that is, one literal that appears complemented in one of them and uncomplemented in the other, then {*S*_*i*_∨*A*_*j*_} is 1 and hence it is ignored since it does not affect a POS formula when multiplied with it. Now, further benefit gained from the above construction is made apparent via the following novel theorem.

Theorem 4 . In the consensus-generation table of Table 3,

(1) there are no absorptions among vertical keys, horizontal keys, and set-aside alterms;
(2) a table entry cannot be absorbed by a table key, but it could be absorbed by another table entry or a set-aside alterm. A set-aside alterm could be absorbed by a table entry;
(3) if a table entry {*S*_*r*_∨*A*_*k*_} is to be ever absorbed by another table entry, then it has an absorbing product for it in the same row *r* or in the same column *k*;
(4) if a table vertical key (*A*_*k*_∨*X*_*m*_) is to be ever absorbed by a table entry, then it has an absorbing product for it in the same column *k*;
(5) if a table horizontal key $\left(S_{r}\vee {\overset{-}{X}}_{m}\right)$ is to be ever absorbed by a table entry, then it has an absorbing product for it in the same row *r*.

In the following, we outline a proof and reflect on the ramifications of Theorem 4.

(1) Each of the conjunctions of vertical keys, that of horizontal keys, and that of set-aside alterms constitutes an absorptive formula. Therefore, there are no absorptions among alterms of such a formula.
(2) A table entry cannot be absorbed by a table key because the former cannot subsume the latter since the former lacks the literal *X*_*m*_ or the literal ${\overset{-}{X}}_{m}$.
(3) Suppose that the table entry {*S*_*r*_∨*A*_*k*_} subsumes (and hence is absorbed by) another table entry {*S*_*i*_∨*A*_*j*_} which lies in a different row (*i* ≠ *r*) and a different column (*j* ≠ *k*). This means that the set of literals of {*S*_*r*_∨*A*_*k*_} is a superset of the set of literals of {*S*_*i*_∨*A*_*j*_} and hence it is a superset of each of the set of literals of *S*_*i*_ and that of *A*_*j*_, and hence {*S*_*r*_∨*A*_*k*_} subsumes both *S*_*i*_ and *A*_*j*_. By construction, {*S*_*r*_∨*A*_*k*_} subsumes both *S*_*r*_ and *A*_*k*_. Now, since {*S*_*r*_∨*A*_*k*_} subsumes the four alterms *S*_*i*_, *A*_*j*_, *S*_*r*_, and *A*_*k*_, it subsumes each of the two alterms {*S*_*i*_∨*A*_*k*_} (which lies in the same column as {*S*_*r*_∨*A*_*k*_}) and {*S*_*r*_∨*A*_*j*_} (which shares the same row as {*S*_*r*_∨*A*_*k*_}). In conclusion, if a general alterm {*S*_*r*_∨*A*_*k*_} is to be ever absorbed by another alterm in the table, then we can find an absorbing alterm for it either in the same row *r* or in the same column *k*.
(4) Now, suppose that the vertical table key (*A*_*k*_∨*X*_*m*_) subsumes (and hence is absorbed by) a table entry {*S*_*i*_∨*A*_*j*_} which lies in a different column (*j* ≠ *k*). This means that the set of literals of (*A*_*k*_∨*X*_*m*_) is a superset of the set of literals of {*S*_*i*_∨*A*_*j*_} and hence it is a superset of each of the set of literals of *S*_*i*_ and that of *A*_*j*_, and hence (*A*_*k*_∨*X*_*m*_) subsumes both *S*_*i*_ and *A*_*j*_. By construction, (*A*_*k*_∨*X*_*m*_) subsumes *A*_*k*_. Now, since (*A*_*k*_∨*X*_*m*_) subsumes the two alterms *S*_*i*_ and *A*_*k*_, it subsumes the alterm {*S*_*i*_∨*A*_*k*_} which lies in the same column as (*A*_*k*_∨*X*_*m*_). In conclusion, if a table vertical key (*A*_*k*_∨*X*_*m*_) is to be ever absorbed by a table entry, then it has an absorbing alterm for it in the same column *k*.
(5) Likewise, it can be shown that if a table horizontal key $\left(S_{r}\vee {\overset{-}{X}}_{m}\right)$ is to be ever absorbed by a table entry, then it has an absorbing alterm for it in the same row *r*.

To change the conjunction of alterms in the whole table (including keys, entries, and set-aside alterms) into an absorptive formula, there is no need to compare every alterm with all other alterms in the whole table. Instead, every remaining table entry not equal to 1 is either absorbed in another in the same row or column of the table or in one of the set-aside alterms or it stays unabsorbed. A vertical table key is either absorbed in a table entry in the same column of the table or it stays unabsorbed. A horizontal table key is either absorbed in a table entry in the same row of the table or it stays unabsorbed. A set-aside alterm is either absorbed in one of the remaining (not equal to 1) table entries or it stays unabsorbed.

In summary, the number of comparisons needed to implement the absorption operation ABS(…) is limited in the worst case to the sum of the following operations:

(1) comparing each remaining table entry not equal to 1 to the alterms with fewer or the same number of literals in (*i*) its row and column of the table, and (*ii*) the set aside alterms;
(2) comparing each vertical table key to the table entries not equal to 1 with fewer or the same number of literals in its column of the table;
(3) comparing each horizontal table key to the table entries not equal to 1 with fewer or the same number of literals in its row of the table;
(4) comparing each of the set-aside alterms to the remaining table entries not equal to 1 with fewer or the same number of literals.

## References

[1] Robinson J. A. (1965). A machine-oriented logic based on the resolution principle. *Journal of the ACM*.

[2] Lee R. C. (1972). Fuzzy logic and the resolution principle. *Journal of the Association for Computing Machinery*.

[3] Mukaidono M. (1982). *Fuzzy Inference of Resolution Style*.

[4] Mukaidono M., Shen Z., Ding L. Fuzzy prolog.

[5] Shen Z., Ding L., Mukaidono M. (1988). A theoretical framework of fuzzy prolog machine. *Fuzzy Computing*.

[6] Shen Z., Ding L., Mukaidono M. Fuzzy resolution principle.

[7] Kim C. S., Lee S. J., Park S. C., Kim D. S. (1993). Fuzzy hyper-resolution: a semantic inference rule with fuzzy concepts. *Korea Fuzzy Mathematics and Systems Society*.

[8] Kim C., Park S., Kim D., Lee S. (1994). A fuzzy hyper-resolution using compensatory operators. *Journal of the Korea Information Science Society*.

[9] Kim C. S., Kim D. S., Park J. S. (2000). A new fuzzy resolution principle based on the antonym. *Fuzzy Sets and Systems*.

[10] Gaines B. R. (1976). Foundations of fuzzy reasoning. *International Journal of Man-Machine Studies*.

[11] Tsukamoto Y. (1979). An approach to fuzzy reasoning method. *Advances in Fuzzy Set Theory and Applications*.

[12] Mizumoto M., Zimmermann H.-J. (1982). Comparison of fuzzy reasoning methods. *Fuzzy Sets and Systems*.

[13] Dubois D., Prade H. (1984). Fuzzy logics and the generalized modus ponens revisited. *Cybernetics and Systems*.

[14] Magrez P., Smets P. (1989). Fuzzy modus ponens: a new model suitable for applications in knowledge-based systems. *International Journal of Intelligent Systems*.

[15] Takagi H., Hayashi I. (1991). NN-driven fuzzy reasoning. *International Journal of Approximate Reasoning*.

[16] Hellendoorn H. (1992). The generalized modus ponens considered as a fuzzy relation. *Fuzzy Sets and Systems*.

[17] Demirli K., Turksen I. B. A review of implications and the generalized modus ponens.

[18] Fodor J. C., Keresztfalvi T. (1995). Nonstandard conjunctions and implications in fuzzy logic. *International Journal of Approximate Reasoning*.

[19] Cordón O., Del Jesus M. J., Herrera F. (1999). A proposal on reasoning methods in fuzzy rule-based classification systems. *International Journal of Approximate Reasoning*.

[20] Yager R. R. (1999). On global requirements for implication operators in fuzzy modus ponens. *Fuzzy Sets and Systems*.

[21] Liu J., Ruan D., Xu Y., Song Z. (2003). A resolution-like strategy based on a lattice-valued logic. *IEEE Transactions on Fuzzy Systems*.

[22] Igel C., Temme K.-H. (2004). The chaining syllogism in fuzzy logic. *IEEE Transactions on Fuzzy Systems*.

[23] Tick J., Fodor J. Fuzzy implications and inference processes.

[24] Blake A. (1937). *Canonical expressions in boolean algebra [Ph.D. thesis]*.

[25] Brown F. M. (1990). *Boolean Reasoning: The Logic of Boolean Equations*.

[26] Gregg J. (1998). *Ones and Zeros: Understanding Boolean Algebra, Digital Circuits, and the Logic of Sets*.

[27] Rushdi A. M., Al-Shehri A. S. (2002). Logical reasoning and its supporting role in the service of security and justice. *Journal of Security Studies*.

[28] Rushdi A. M., Ba-Rukab O. M. (2007). Some engineering applications of the modern syllogistic method. *SEC7 Paper*.

[29] Rushdi A. M. (2008). The modern syllogistic method as a tool for engineering problem solving. *Journal of Qassim University: Engineering and Computer Sciences*.

[30] Rushdi A. M., Barukab O. M. (2009). An exposition of the modern syllogistic method of propositional logic. *Umm Al-Qura University Journal: Engineering and Architecture*.

[31] Rushdi A. M., Ba-Rukab O. M. (2008). Powerful features of the modern syllogistic method of propositional logic. *Journal of Mathematics and Statistics*.

[32] Rushdi A. M. A., Ba-Rukab O. M. (2014). Switching-algebraic analysis of relational databases. *Journal of Mathematics and Statistics*.

[33] Rushdi A. M., BaRukab O. M. (2014). Map derivation of the closures for dependency and attribute sets and all candidate keys for a relational database. *Journal of King Abdulaziz University: Engineering Sciences*.

[34] Chang C. L., Lee R. C. (1973). *Symbolic Logic and Mechanical Theorem Proving*.

[35] Davis M., Putnam H. (1960). A computing procedure for quantification theory. *Journal of the ACM*.

[36] Copi I., Cohen C. (2010). *Introduction to Logic*.

[37] Klenk V. (2013). *Understanding Symbolic Logic*.

[38] Atanassov K. (1988). Two variants of intuitionistic fuzzy propositional calculus.

[39] Ciftcibasi T., Altunay D. Fuzzy propositional logic and two-sided (intuitionistic) fuzzy propositions.

[40] Atanassov K., Gargov G. (1998). Elements of intuitionistic fuzzy logic. Part I. *Fuzzy Sets and Systems*.

[41] Atanassov K. T. (1999). *Intuitionistic Fuzzy Sets*.

[42] Cornelis C., Deschrijver G., Kerre E. E. Classification of intuitionistic fuzzy implicators: an algebraic approach.

[43] Atanassov K. On eight new intuitionistic fuzzy implications.

[44] Atanassova L. (2009). A new intuitionistic fuzzy implication. *Cybernetics and Information Technologies*.

[45] Atanassov K. T. On intuitionistic fuzzy negations and law for excluded middle.

[46] Wan S.-P., Li D.-F. (2014). Atanassov's intuitionistic fuzzy programming method for heterogeneous multiattribute group decision making with atanassov's intuitionistic fuzzy truth degrees. *IEEE Transactions on Fuzzy Systems*.

[47] Wang J.-Q., Zhang H.-Y. (2013). Multicriteria decision-making approach based on atanassov's intuitionistic fuzzy sets with incomplete certain information on weights. *IEEE Transactions on Fuzzy Systems*.

[48] Papageorgiou E. I., Iakovidis D. K. (2013). Intuitionistic fuzzy cognitive maps. *IEEE Transactions on Fuzzy Systems*.

[49] Zadeh L. (1965). Fuzzy set. *Information and Control*.

[50] Zadeh L. A. (1968). Fuzzy algorithms. *Information and Control*.

[51] Marinos P. N. (1969). Fuzzy logic and its application to switching systems. *IEEE Transactions on Computers*.

[52] Lee R. C., Chang C.-l. (1971). Some properties of fuzzy logic. *Information and Computation*.

[53] Zadeh L. A. (1988). Fuzzy logic. *Computer*.

[54] Klir G. J., Folger T. A. (1988). *Fuzzy Sets, Uncertainty, and Information*.

[55] Ross T. J. (2010). *Fuzzy Logic with Engineering Applications*.

[56] Muroga S. (1979). *Logic Design and Switching Theory*.

[57] Tison P. (1967). Generalization of consensus theory and application to the minimization of boolean functions. *IEEE Transactions on Electronic Computers*.

[58] Cutler R. B., Kinoshita K., Muroga S. (1979). *Exposition of Tison's Method to Derive All Prime Implicants and All Irredundant Disjunctive Forms for a Given Switching Function*.

[59] Loui M., Bilardi G. (1982). The correctness of Tison's method for generating prime implicants.

[60] Klir G. J., Marin M. A. (1969). New considerations in teaching switching theory. *IEEE Transactions on Education*.

[61] Kalish D., Montague R. (1964). *Logic. Techniques of Formal Reasoning*.

[62] Rushdi A. M., Zarouan M., Alshehri T. M., Rushdi M. A. (2015). The incremental version of the modern syllogistic method. *Journal of King Abdulaziz University: Engineering Sciences*.

[63] Rushdi A. M., Alshehri T. M., Zarouan M., Rushdi M. A. (2015). Utilization of the modern syllogistic method in the exploration of hidden aspects in engineering ethical dilemmas. *Journal of King Abdulaziz University: Computers and Information Technology*.

[64] Rushdi A. M., Al-Yahya H. A. (2000). Derivation of the complete sum of a switching function with the aid of the variable entered karnaugh map. *Journal of King Saud University*.

[65] Rushdi A. M. A., Albarakati H. M. (2012). The inverse problem for Boolean equations. *Journal of Computer Science*.

[66] Kean A., Tsiknis G. (1990). An incremental method for generating prime implicants/implicates. *Journal of Symbolic Computation*.

[67] Rushdi A. M. A., Albarakati H. M. (2014). Construction of general subsumptive solutions of Boolean equations via complete-sum derivation. *Journal of Mathematics and Statistics*.

[68] Slagle J. R., Chang C. L., Lee R. C. (1970). A new algorithm for generating prime implicants. *IEEE Transactions on Computers*.

[69] Hwa H. R. (1974). A method for generating prime implicants of a boolean expression. *IEEE Transactions on Computers*.

[70] Reusch B. (1975). Generation of prime implicants from subfunctions and a unifying approach to the covering problem. *IEEE Transactions on Computers*.

[71] Coudert O., Madre J., Knight T., Savage J. (1992). A new method to compute prime and essential prime implicants of boolean functions. *Advanced Research in VLSI and Parallel Systems*.

[72] Rushdi A. M., Al-Yahya H. A. (2000). A boolean minimization procedure using the variable-entered karnaugh map and the generalized consensus concept. *International Journal of Electronics*.

[73] Rushdi A. (2001). Prime-implicant extraction with the aid of the variable-entered karnaugh map. *Umm Al-Qura University Journal: Science, Medicine and Engineering*.

[74] Alexe G., Alexe S., Crama Y., Foldes S., Hammer P. L., Simeone B. (2004). Consensus algorithms for the generation of all maximal bicliques. *Discrete Applied Mathematics*.

[75] Ślęzak D. (2006). Association reducts: boolean representation. *Rough Sets and Knowledge Technology*.

[76] Pawlak Z., Skowron A. (2007). Rough sets and boolean reasoning. *Information Sciences*.

[77] Crama Y., Hammer P. L. (2011). *Boolean Functions: Theory, Algorithms, and Applications*.

